# Virtual Simulations as Preparation for Lab Exercises: Assessing Learning of Key Laboratory Skills in Microbiology and Improvement of Essential Non-Cognitive Skills

**DOI:** 10.1371/journal.pone.0155895

**Published:** 2016-06-02

**Authors:** Guido Makransky, Malene Warming Thisgaard, Helen Gadegaard

**Affiliations:** 1 University of Southern Denmark, Odense, Denmark; 2 University of Glasgow, Glasgow, Scotland; Laurentian, CANADA

## Abstract

**Objective:**

To investigate if a virtual laboratory simulation (vLAB) could be used to replace a face to face tutorial (demonstration) to prepare students for a laboratory exercise in microbiology.

**Methods:**

A total of 189 students who were participating in an undergraduate biology course were randomly selected into a vLAB or demonstration condition. In the vLAB condition students could use a vLAB at home to ‘practice’ streaking out bacteria on agar plates in a virtual environment. In the demonstration condition students were given a live demonstration from a lab tutor showing them how to streak out bacteria on agar plates. All students were blindly assessed on their ability to perform the streaking technique in the physical lab, and were administered a pre and post-test to determine their knowledge of microbiology, intrinsic motivation to study microbiology, and self-efficacy in the field of microbiology prior to, and after the experiment.

**Results:**

The results showed that there were no significant differences between the two groups on their lab scores, and both groups had similar increases in knowledge of microbiology, intrinsic motivation to study microbiology, as well as self-efficacy in the field of microbiology.

**Conclusion:**

Our data show that vLABs function just as well as face to face tutorials in preparing students for a physical lab activity in microbiology. The results imply that vLABs could be used instead of face to face tutorials, and a combination of virtual and physical lab exercises could be the future of science education.

## Introduction

Laboratory exercises are an essential part of many science courses, because they provide a practical extension of the concepts taught, letting students acquire practical skills through experience [1,2]. They give the students the possibility to get a deeper and physically rooted understanding of the content, supporting learning in scientific fields [3,4]. According to Bernhard (2010) a fundamental purpose of the laboratory is to develop holistic, conceptual knowledge [5].

An important predictor of the success in lab exercises is the amount of preparation students have had before performing the lab experiments relevant to a certain topic. Being able to focus on the experiment at hand and comprehend its critical features is an important prerequisite for successful learning [5]. According to cognitive load theory it is necessary to direct cognitive resources towards the relevant activity, to learn from it [6]. If one instead directs ones cognitive resources towards irrelevant activities, learning will be obstructed. To get the best possible learning outcome from the lab, it is therefore essential that students know the basics of working in a lab, and have a fundamental understanding of how to use the different equipment, as well as basic cognitive skills relevant to the work. If not, they will waste valuable time and cognitive resources trying to understand how to use the lab and solve technical issues, and therefore miss learning as much as possible from the actual experiment. Therefore, it is evident that thorough preparation before entering the lab is crucial if one wishes the students to gain as much learning from the lab work as possible.

The guidance that students receive to instruct their lab activities is recognized as being essential to their learning [3]. Typically, teachers prepare their students to work in the lab by giving face to face tutorials. Our experience is that this is usually done in small groups at the start of a teaching lab session, with staff to student ratios in the region of 1:16. This is effective in equipping the students with the sufficient amount of basic knowledge and smaller, more informal teaching groups can promote cooperative learning and allow meaningful student inquiry [3], but there are some important drawbacks. Preparing students for lab exercises in this way can be time consuming and expensive. In addition, teachers have a limited amount of time for each tutorial and individual student, making it difficult to ensure that every student gains the required experience and information, and that relevant individual differences are appropriately taken into account. A student who finds the basics of the lab challenging might not be able to get sufficient help, because of these limits. This results in classes of students with widely different foundations of basic knowledge, making the actual experiment harder for the teacher to oversee and reducing the quality of interaction between teacher and student.

As an alternative to hands on tutorials, virtual laboratory simulations (vLAB) represent an exciting new way of preparing students for hands on exercises, such as laboratory work [7–9], and various computer based technologies are now being recognized as enabling reform of laboratory teaching practice away from the traditional didactic model [10]. Previous research has shown that using instructional videos before laboratory exercises constituted an effective tool in standardizing teaching and promoting successful outcomes in subsequent physical laboratory exercises [11]. In a review of research on technology-assisted school science laboratories, Wang et al. (2014) found that simulations were often used as preparation for physical laboratory work, enhancing learning and performance by providing students with essential concepts and principles prior to the physical lab [12]. Giving students their first encounter with laboratory work in a virtual environment lets them explore the basics in a safe and time efficient manner. The vLABs can be played by the student at a time and location of their choice adding a degree of flexibility to their learning, [13] as well as providing a learning experience accommodating a variety of individual differences. They can use as much time as they need and use the simulation in a way that is concordant with their specific learning strategy. The provision of high quality, stimulating preparatory work for students should allow easier provision of inquiry-based teaching approaches in laboratory classes, the value of which is undisputed but not yet the paradigm in teaching practice [14]. Students with sufficient basic skills and knowledge obtained during preparation, will not need to use their cognitive resources on dealing with practical issues [6], and can instead direct their cognitive resources at the inquiry at hand, solving experimental issues and problems.

An important note to the use of simulations is that they will only serve as a tool for preparation, not as a replacement for the physical lab in its entirety. This is important because even though the simulations afford many advantages, they do not provide every affordance the physical labs do; they are unable to provide the same physical experiences and tactile information as the physical lab [1], which represent critical ingredients in lab learning. In support of the combination of virtual simulations with physical lab work, the review of research on technology-assisted school science laboratories, mentioned earlier, reported that across various studies a consistent conclusion was that the combination of the two teaching methods creates a greater learning effect [12].

VLABs are increasingly being used in educational contexts [2,15]. They are being used in several areas of health care for professional skills training and assessment [16–18], as well as chemistry courses [19,20], biotech education [21], genetics [22] and a wide variety of other fields [2]. As early as 1992 a study showed the value of interactive computer based learning, reporting equally good efficacy of the computer based learning method, compared to the conventional laboratory approach, as well as highlighting the method as highly time-efficient [23]. Utilizing inquiry based learning [24] and dynamic visualizations [25], vLABs provide an extensive learning platform that not only add to student learning, but can also increase their motivation to learn and improve their self-efficacy appraisals. For instance, a study by Bonde and colleagues (2014), found a significant increase in both learning, and motivation when a vLAB was compared with a traditional lecture [21]. Furthermore, Makransky and colleagues (2016) found that a vLAB lead to a significant increase in learning, motivation, and self-efficacy for weak and strong students alike [22]. Another study by Polly, Marcus, Maguire, Belinson and Velan (2015), concluded that students found a vLAB at least as helpful for learning as a real lab, especially in relation to learning application of laboratory methods [26]. Similarly, Weller (2004), found a subjective increase in students competency with the learnt material after a simulation-based workshop, with students rating the simulations workshop highly [27]. In a critical review of the literature, Smetana and Bell (2012) summarized that simulations can be comparable, if not more effective, than more traditional educational practices, depending on how they are used, recommending simulations be used as a supplement to other instructional methods [13]. Another review by De Jong et al. (2013) concluded that the need for investigation in science courses, can be fulfilled by both virtual and physical investigations, highlighting that virtual experiments provide a unique opportunity to observe otherwise unobservable phenomena [1]. They also recommend a combination of virtual and physical laboratories, in that they offer advantages that neither one can provide in itself.

At this time, most of the existing research on the applicability of vLABs has focused on the evaluation of vLABs compared to lectures. Some research has already investigated the utility of combining virtual simulations and physical lab work [12] and even using the simulations as preparation for physical lab work [11]. However, there still is much to be learned about using simulations as tools for preparing students for physical lab work, and more research is needed, that directly investigates the viability of using virtual laboratory simulations as preparation material for physical lab exercises. This need is particularly true when it comes to investigating the possibility of using vLABs to prepare students for laboratory exercises, as an alternative to face to face tutorials, given the time and resource requirements of the latter. It is also important that this research focus on outcomes such as non-cognitive skills in the form of motivation and self-efficacy in addition to cognitive skills such as knowledge, since there is growing evidence suggesting that these factors are just as, if not more, important than cognitive skills in academic success and play important roles in relation to students’ academic achievement, drop-out rates, as well as professional results after obtaining a degree [28–31].

The aim of this paper is thus to investigate if a vLAB can be used to replace a face to face tutorial to prepare students for a laboratory exercise. More specifically, we investigate if a group of students who prepare for a laboratory exercise in microbiology by using a vLAB have comparable outcomes to a group who are given a face to face tutorial by an experienced teacher. In extension of this aim two specific research questions are posed:

**Research question 1.** Do students who use a vLAB have equally high lab scores, compared to a group of students who are given a face to face tutorial as preparation for a laboratory exercise?
**Research question 2.** Does a vLAB, followed by a physical lab exercise in microbiology, lead to an increase in a) student knowledge of microbiology, b) intrinsic motivation to study microbiology, c) self-efficacy in the field of microbiology, and is this increase comparable to the use of a face to face tutorial followed by a physical lab exercise?

## Materials and Methods

### Sample

The sample consisted of 210 students from the University of Glasgow who were participating in an undergraduate microbiology course. Of those 189 agreed to participate. There were 131 females and 68 males and they had an average age of 20.2 (see S1 Data. Data used in the study). The project was fully explained to students before they were given a written information sheet describing the research aims, and written consent was gathered from voluntary participants. The study was approved by the College of Medical, Veterinary & Life Sciences Ethics Committee at the University of Glasgow (October 2015).

### Procedure

Students were randomly selected into a face to face lab tutorial (referred to as demonstration) or virtual simulation (referred to as vLAB) group. All students were then given a pre-test to determine their baseline knowledge of microbiology, intrinsic motivation to study microbiology, and self-efficacy in the field of microbiology.

The demonstration group made up of 94 students was split into 3 lab tutorial groups of approximately 31 students per group. These groups attended a teaching laboratory, where they were given a demonstration from a lab tutor (about 15 students/tutor) in a ‘traditional’ manner showing them how to streak out bacteria on agar plates to isolate single colonies. The tutor also described the principles of using selective and differential culture media. The material taught in this session was designed to reflect, as much as possible, the learning that students were hoped to achieve in the lab. Immediately following the demonstration, students were given a written sheet of instructions describing their task. Students performed an exercise in streaking out bacteria to achieve single isolated colonies, free from contamination, followed by the post-test. This group of students was also given access to the Labster simulation after completing the physical lab and the post-test to ensure that no students were disadvantaged due to the study.

The vLAB condition had 95 students and involved using a virtual laboratory simulation developed by the simulation-focused company Labster. Students in this group were asked to complete the simulation in their own time. In the simulation students were set the task of isolating a bacterium that had caused an incidence of food poisoning. They were introduced to the principles of using selective and differential culture media in microbiology, and given repeated opportunities to ‘practice’ streaking out bacteria on agar plates, incubate them, and culture isolated colonies free from contamination. This is a key technique in microbiology lab practice, and had not been previously taught in the class (see S1 Video of bacterial isolation vLAB simulation). Approximately 1 week after these students first had access to the vLAB, they were asked to attend a physical lab to assess their learning. Data showed that students accessed the simulation spread out across the entire week with some students using the simulation right away, while others used it right before the physical lab activity. In the physical lab, students were asked to perform the same procedure as students from the demonstration group, and were given the same written instructions to follow without any further teaching support.

A total of 87% of the students from both groups used the Labster vLAB. Students had the ability to return to the vLAB to repeat their lab practice freely, however most students (80%) only used the simulation one time, 16% used it two times, 3% used it three times, and 1% used it four times. The median time used on the vLAB was 56 minutes and ranged from 14 minutes to more than several days since students did not have to log out of the simulation while not using it.

The performance of the vLAB and Demonstration groups in the teaching lab were blind-marked by one tutor. Their ability to perform the technique was assessed on a scale from 1–5. A marking rubric was composed and used to analyse the lab performance. The ideal streak plate (5 marks) had multiple, well-resolved single colonies, with the full area of the petri dish utilised and no obvious contaminants. Marks were deducted for less than ideal streak plates (1 mark when only one or two well-resolved single colonies attained, when streaking didn’t cover the entire petri dish, or one or two contaminant colonies present; 2 marks when there were no single colonies, streaking used only a small area of the petri dish or 3–5 contaminant colonies). No marks were awarded if there was no growth at all on the plate, or the plate was heavily contaminated. Students were also given a post-test to reassess their knowledge of microbiology, motivation to study microbiology, and self-efficacy in the field of microbiology.

### Main Outcome Measures

Knowledge of microbiology was assessed with 10 multiple choice questions (see S1 Table. List of questions used in the study). Motivation was assessed using 5 questions from the Interest/Enjoyment Scale from the Intrinsic Motivation Inventory [32] (see S1 Table. List of questions used in the study). Self-efficacy for learning microbiology was assessed using 8 questions from the Motivated Strategies for Learning Questionnaire [33] (see S1 Table. List of questions used in the study).

Students indicated their responses to the motivation and self-efficacy items using a 5-point Likert scale (1 = *Strongly disagree*, 5 = *Strongly agree*). The IBM SPSS software package was used to investigate the two research questions in the study, and the Rumm program was used to assess the validity of the scales within the framework of Item Response Theory [34]. Data was collected in Surveymonkey and within the Labster program.

## Results

**Fig 1:**
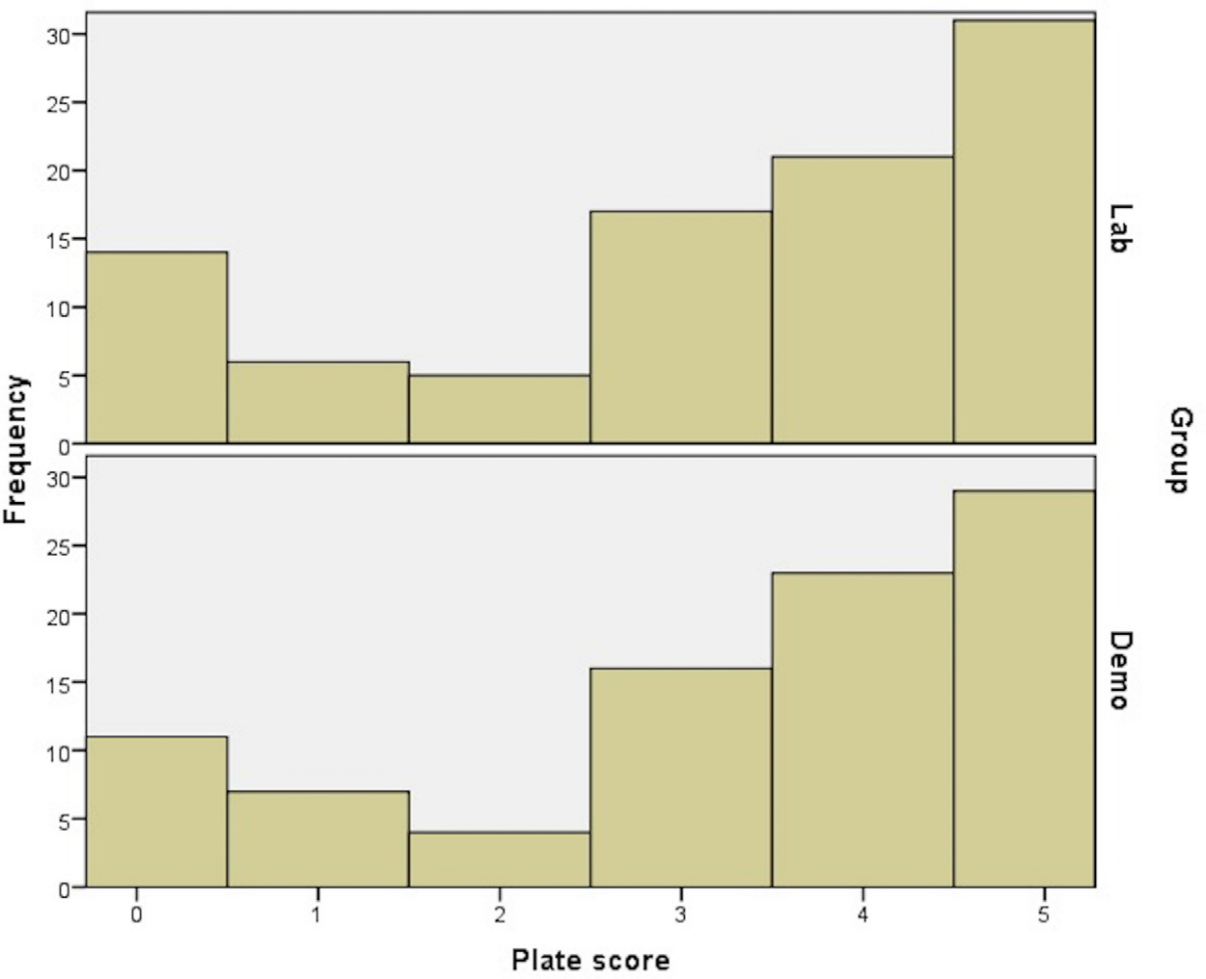
Distribution of the plate scores for the bacteria isolation task for the vLAB and demonstration groups.

Research question 1 was investigated by conducting an independent samples t-test to assess if the lab scores were significantly different across the two conditions. The two groups had almost identical average scores from their plate exercise (vLAB group mean = 3.26; demonstration group mean = 3.33) and an independent samples t-test showed that the difference was not significant *t*_(182)_ = 0.762, *p* = .303; *d* = 0.04 (see Fig 1 for a graphical representation of the results).

Prior to investigating research question 2, we examined if the vLAB and demonstration groups had significantly different pre-test scores on the three outcome variables. The results showed that the groups did not have significantly different scores on knowledge of microbiology, intrinsic motivation, or self-efficacy, so it was not necessary to use these as covariates in the analyses (see Table 1). Furthermore, we inspected the validity and reliability of the three outcome measures (intrinsic motivation, self-efficacy, and knowledge of biology) by checking the fit of the items to the Partial Credit Rasch model (PCM) [35] within the framework of Item Response Theory. The results supported the construct validity of the five item intrinsic motivation measure based on good general fit to the Rasch model in the pre-test (χ2 [10] = 7.64, *p* = .68) and the post-test (χ2 [10] = 4.98, *p* = .89), furthermore, all items fit the model and there was no local-dependence which indicated no redundancy between the items (see [36–38] for more information about criteria used when assessing fit to the PCM). The scale also had good reliability with a Cronbach’s alpha of 0.84 in the pre-test and 0.98 in the post-test. Similarly, the eight item self-efficacy scale had acceptable fit to the PCM in the pre-test (χ2 [16] = 21.32, *p* = .17) and the post-test (χ2 [16] = 10.66, *p* = .83), and good reliability with a Cronbach’s alpha of 0.87 in the pre-test and 0.91 in the post test. The 10 item knowledge scale had good fit to the PCM in the pre-test (χ2 [20] = 14.64, *p* = .80) and the post-test (χ2 [20] = 25.14, *p* = .20), but very low reliability with a Cronbach’s alpha of 0.45 in the pre-test and 0.58 in the post-test. These results support the validity and reliability of the intrinsic motivation and self-efficacy scales but the results based on the knowledge scale should be interpreted cautiously due to the low reliability of the scale.

**Table 1:** Mean scores for the outcome variables in the study in the pre- and post- test for the vLAB and Demonstration groups. Note: Standard deviation is reported in parentheses.

Scale	vLAB		Demonstration	
	Pre-test	Post-test	Pre-test	Post-test
Lab scores		3.26 (1.77)		3.33 (1.72)
Knowledge	5.95 (1.95)	6.89 (2.16)	5.91 (1.83)	6.98 (1.86)
Motivation	4.06 (0.56)	4.18 (0.53)	4.06 (0.55)	4.11 (0.58)
Self-efficacy	3.41 (0.58)	3.55 (0.58)	3.49 (0.54)	3.67 (0.58)

**Fig 2:**
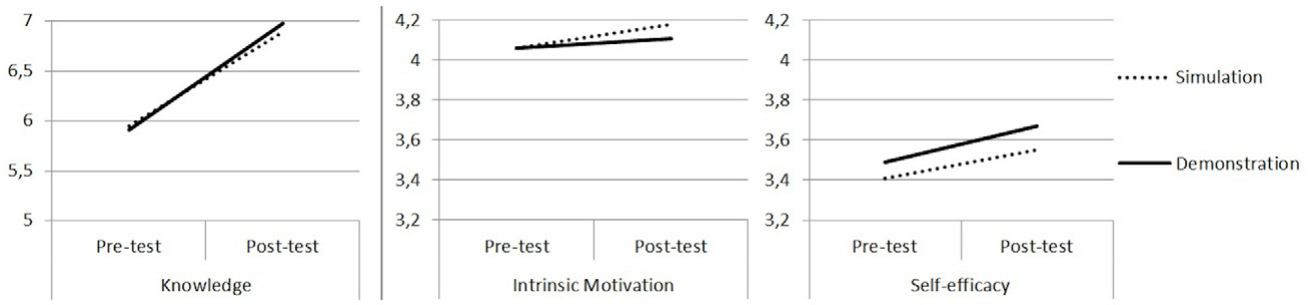
Visual representation of the change in knowledge, intrinsic motivation and self-efficacy for the vLAB and demonstration groups.

Research question 2 was entered into a 2 group (vLAB/demonstration) by 2 time points (pre/post-test) mixed model ANOVA for the three outcome measures to assess whether pre/post-test changes were different across the two groups. The results were similar for all three outcome variables, namely there was not a significant group by time interaction for knowledge of microbiology (*F*_(1)_ = .180, *p* = .672), intrinsic motivation (*F*_(1)_ = 1.364, *p* = .245), or self-efficacy (*F*_(1)_ = .502, *p* = .480). Since there were no between subject effects, paired samples t-tests were run for each group to assess if there was a significant increase in knowledge of microbiology, intrinsic motivation to study microbiology, and self-efficacy in the field of microbiology from the pre- to post-test. For the vLAB group, the paired samples *t*-tests showed a significant increase in knowledge of microbiology from a mean of 5.95 correct on the pre-test, to 6.88 correct on the post-test, *t*_(76)_ = 4.73, *p* < .001; *d* = 0.45. A significant increase was also found in intrinsic motivation for microbiology from a mean of 4.06 on a scale from 1 to 5 in the pre-test to 4.18 in the post test, *t*_(76)_ = 2.80, *p* = .006; *d* = 0.22. Finally, the group had a significant increase in self-efficacy in microbiology from a mean of 3.41 in the pre-test to 3.55 in the post test *t*_(76)_ = 3.62, *p* = .001; *d* = 0.24 (see Fig 2).

The demonstration group also had a significant increase in knowledge and self-efficacy but a non-significant increase in intrinsic motivation. Paired samples *t*-tests showed a significant increase in knowledge in microbiology from a mean of 5.91 correct on the pre-test to 6.98 correct on the post-test, *t*_(89)_ = 5.24, *p* < .001; *d* = 0.58. There was not a significant increase in intrinsic motivation from a mean of 4.06 on a scale from 1 to 5 in the pre-test to 4.11 in the post test, *t*_(84)_ = 1.58, *p* = .119; *d* = 0.09. Finally, a significant increase in self-efficacy in microbiology from a mean of 3.49 in the pre-test to 3.67 in the post test *t*_(84)_ = 4.24, *p* < .001; *d* = 0.32 was found.

## Discussion

Virtual laboratory simulations (vLABs) have shown very promising results when evaluated compared to more traditional educational methods, however few studies have evaluated a vLAB as preparation material for a physical laboratory exercise. In this study we investigated if a group of students who prepared for a laboratory exercise in microbiology by using a vLAB had comparable outcomes to a group who were given a face to face tutorial by an experienced teacher.

The first research question explored whether or not students using a vLAB would achieve equally high lab scores, compared to students given a face to face tutorial. The results showed that there were no significant differences between the two groups. That is, the group of students who were given the vLAB as preparation for the physical lab exercise performed equally well on the laboratory exercise as the group of students who were given a face to face tutorial. The second research question investigated if the vLAB followed by a physical lab exercise in microbiology, lead to an increase in student knowledge of microbiology, intrinsic motivation to study microbiology, and self-efficacy in the field of microbiology, and if this increase was comparable to the use of a face to face tutorial followed by a physical lab exercise. Results showed that there was not a significant group by time interaction for knowledge of microbiology, intrinsic motivation or self-efficacy from the pre- to post-test. Both groups had significant increases in knowledge of microbiology, and self-efficacy in microbiology, and the group who used the vLAB had a significant increase in intrinsic motivation to study microbiology.

The results suggest that a vLAB can successfully be used as preparation material for a physical lab activity. This is very interesting because it broadens the possible application of vLABs within education, as a way to alleviate the cognitive load of working in a real lab, by giving the students an effective way to prepare and gain the basic knowledge and cognitive skills beforehand, thereby allowing them to direct all their cognitive resources towards the relevant activity in the real lab. This is an important breakthrough in this area of research, because the positive findings show that vLABs can be used as a compliment to real lab work, and not just as a substitute for traditional educational methods.

The median simulation time was 56 minutes, and replaced human-tutor instruction that typically takes 15–20 min. With the tutor needing to replicate their teaching as many times as necessitated by size of class and lab groups, in our class, the simulation could replace 15 replicates of human-tutor instruction; approximately 4–5 hours total staff contact time. The simulation however covered much more than what would have been given in the human-tutor prep; it allowed practice of the technique and instant observation of the result (which would have required 2 days incubation in ‘real-time’for bacterial colonies to grow, materials and technical support). The student was able to practice as many times as they wished without any of these constraints.

The finding that both groups of students had significant increases in knowledge of microbiology, self-efficacy in microbiology, and the group who used the vLAB had a significant increase in intrinsic motivation to study microbiology, supports existing evidence that practical exercises and activities that activate students lead to positive cognitive and non-cognitive outcomes. Previous research has found that vLABs could increase student knowledge [21,26]. Although increasing cognitive skills like knowledge is an important outcome, and often the main objective of an educational activity, increases in non-cognitive skills such as motivation and self-efficacy could prove to have even more significant long term impact [22]. The importance of this is supported in the growing evidence suggesting that non-cognitive skills play an essential role in academic success [28–31]. Intrinsic motivation has been shown to be an important mediator for short and long term educational outcomes [39]. Furthermore, increasing self-efficacy is important because there is evidence that belief in one’s ability to succeed can lead to greater educational and life outcomes than ones actual ability [40]. This is because students with a high level of self-efficacy are more likely to participate, work harder, persist longer, and choose more challenging tasks and goals. In light of this, the results of the present study demonstrate that the vLAB is not only suited to replace a face to face tutorial with regards to cognitive outcomes, but that it also caters to the important non-cognitive aspects of learning when used in combination with a physical laboratory exercise.

### Methodological considerations and future research

In this study we compared a vLAB to a face to face tutorial; future research should investigate what characteristics a vLAB should have to make the preparation optimal and if there are different characteristics needed for different types of students. Future research should also investigate the generalizability of the results of this study. In the current study the vLAB was used to prepare students for a fairly simple physical lab activity. More research is needed to investigate the usefulness of the results in a broader context where the effectiveness of vLABS are compared to demonstrations for more complex laboratory techniques, in different subject areas, and across different types of students.

Although the current results provide initial evidence that vLABs can be used to prepare students for physical laboratory exercises by comparing a group of students who prepared for the physical laboratory with a vLAB compared to a face to face tutorial, future research should include additional conditions where students are provided with introduction material through a different platform. An example could be to introduce a third condition where students are given a video introduction of the lab activity.

Future research should also investigate long term effects of using the vLABs for the outcome measures used in this study. Although previous research [21] has found that knowledge gains were retained 40 days after having used a vLAB, there is less evidence of whether gains in self-efficacy or motivation due to a vLAB are stable over a longer period of time. Therefore, future research should assess if the gains in self-efficacy and motivation are maintained by assessing these variables again a few weeks after having used the vLAB. Finally, one of the limitations in this study was the low reliability of the knowledge scale that was used. Therefore, future research should attempt to replicate the results in this study with longer scales, and across different domains.

## Conclusions

The results of the study show that a vLAB functioned just as well as a face to face tutorial in preparing students for a physical lab activity in microbiology. Furthermore, the use of a vLAB or face to face tutorial combined with the physical lab activity was found to lead to significant gains in student knowledge as well as self-efficacy. Also, the combination of the vLAB and physical lab activity lead to increases in intrinsic motivation. The positive results of combining vLABs followed by physical lab activities could thus lead to multiple benefits because they may be able save teaching resources, allow students to master material at their own pace, and can lead to positive long-term consequences for developing professionals that go beyond the specific knowledge that is obtained in a single learning session.

## Supporting Information

S1 Data Data used in the study. (SAV)

S1 Video Video of bacterial isolation vLAB simulation. (MOV)

S1 Table List of questions used in the study. (DOCX)
