# Supplementary material for: Virtual Simulations as Preparation for Lab Exercises: Assessing Learning of Key Laboratory Skills in Microbiology and Improvement of Essential Non-Cognitive Skills
Source: PLoS One. 2016 Jun 2;11(6):e0155895. doi: 10.1371/journal.pone.0155895 (PMC4890735; doi:10.1371/journal.pone.0155895)
Supplement: S1 Table — (DOCX) [file pone.0155895.s003.docx]

**S1 Table. List of questions used in the study**

**Intrinsic Motivation (5 items)**

*Response options: Completely disagree, Disagree, Neither agree nor disagree, Agree, Completely Agree*

- I enjoy working with Microbiology
- Microbiology activities are fun to perform
- Microbiology is boring
- Microbiology does not hold my attention at all
- I would describe Microbiology as very interesting

**Self-efficacy (8 items)**

*Response options: Completely disagree, Disagree, Neither agree nor disagree, Agree, Completely Agree*

- I am confident and can understand the basic concepts of Microbiology
- I am confident that I understand the most complex concepts related to Microbiology
- I am confident that I can do an excellent job on the assignments and tests in the Microbiology exercises
- I expect to do well in Microbiology
- I am certain that I can master the skills being taught in Microbiology
- I believe I will receive an excellent grade in Microbiology
- I’m certain I can understand the most difficult material presented in this course
- Considering the difficulty of this course, the teacher, and my skills, I think I will do well in the class

**Knowledge of microbiology (10 items)**

*The cursive response in bold is the correct answer*

**An unspecific growth medium**

- ***Will support the growth of a wide range of microorganisms***
- Must be incubated at 37°C
- Will contain selective agents
- Will contain differential agents

**The optimal growth temperature of a microorganism**

- ***Usually reflects the physical conditions of the organism’s natural environment***
- Is the highest temperature at which a microorganism can replicate
- Is the median point between the lowest and highest temperature thresholds at which a microorganism can replicate
- Varies depending on the laboratory culture conditions that are set

**Isolated bacterial colonies**

- ***Are clonal populations that arise from a single cell***
- Are usually larger as they have more space to spread
- Are genetically diverse micro-populations that arise from a single cell
- Result from restrictive growth conditions

**Streaking agar plates to achieve isolated colonies**

- ***Requires systematic thinning of bacteria over an agar plate to the point of single cells***
- Requires the wire loop to be sterilised as part of good Health and Safety practice
- Requires the wire loop to be sterilised to pick up a single-cell inoculum
- Requires a selective and differential agar

**A differential agar**

- ***Differentiates between bacterial phenotypes whose growth it can support***
- Selects for the growth of specific microorganisms
- Differentiates between Gram +ve and Gram –ve bacteria
- Will restrict the growth of lactose fermenting bacteria

**A selective agar**

- ***Will be made from one of many agar recipes able to restrict the growth of specific*** *microorganisms*
- Will allow us to observe bacterial colonies restricted by the given growth conditions
- Often indicates the fermentation abilities of microorganisms
- Requires a pH indicator to be added to the agar growth medium

**Instruction given before the following questions were asked:**

***Read the following description of MSA before answering the questions under:*** *Mannitol Salt Agar (MSA) contains mannitol (a sugar) and a high level of salt that limits the growth of many organisms. It is a clear pink colour and contains a pH inidcator that will turn yellow when acidic.*

**In MSA, mannitol is**

- the selective agent
- ***the differential agent***
- a carbon source
- a pH buffer

**The selective constituent in MSA is**

- mannitol
- acidic pH
- ***salt***
- what allows us to differentiate between organisms that can grow on MSA

***Staphylococcus aureus* and *Staphylococcus epidermidis* can both tolerate the salt level in MSA. *S. aureus* alone is able to ferment mannitol. How would you expect their growth to appear on MSA?**

- both colony types will be yellow
- ***S. aureus yellow, S. epidermidis pink***
- S. aureus pink, S. epidermidis yellow
- both colony types will be pink

**The role of the pH indicator is**

- to ensure the correct pH of the agar is maintained
- to select organisms that can tolerate an acidic pH
- ***to allow differentiation between organisms that can ferment mannitol and those which cannot***
- to select organisms that can tolerate high salt levels
